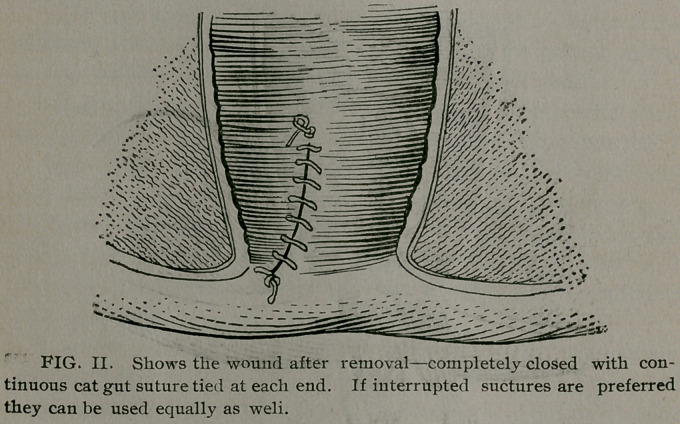# An Operation for Hemorrhoids Practically Painless and Bloodless

**Published:** 1907-10

**Authors:** R. R. Kime

**Affiliations:** Atlanta, Ga.


### AN OPERATION FOR HEMORRHOIDS PRACTICALLY PAINLESS AND BLOODLESS.*

By R. R. Kime, M. D., Atlanta, Ga.

The only apology I have for presenting so trite a subject is the common existence of the disease and the pain the patient so frequently suffers after the usual operation for hemorrhoids.

It is not only the duty of the surgeon to do successful work but prevent his patient suffering after operation when possible.

The most of the operations for this condition have objectionable features and do not fulfil the requirements of ideal surgery.

Some produce unnecessary traumatism, others leave raw surfaces, ulceration, stricture, entail unnecessary loss of blood and most of them produce an undue amount of pain and suffering after operation.

The ideal operation is the one that produces least traumatism, leaves no raw surfaces, crushed, charred or sloughing tissues but clean surfaces properly coaptated to secure rapid primary union with least dangers of infection and with least pain to the patient.

Another feature that promotes comfort of the patient and healing of the wound is a proper preparatory treatment.

Keeping bowels in a laxative condition, flushing them out daily with a boric acid solution when they do not move, introduce into the rectum with a pile pipe an ointment of aristol, zinc oxide, boric acid and vaseline with a gradual dilation of the sphincter muscle with rectal dilators. This line of treatment carried out by the patient at home, relieves many cases and prepares the others for operation by putting rectum in a healthier condition and lessning irritable condition of the sphincter muscle.

---

*Read by title before the Southern Med. & Surg. Asso. Sept. 25-27, 1907, Birmingham, Ala.

It is well to diet all such cases to relieve auto-intoxication and irritation of rectum, and at the same time give such remedies as will improve digestion and tend to disinfect alimentary canal. Before operating, limit diet, exclude milk and food with much residue, moving bowels well day previous so as not to interfere at time of operation, cleanse, prepare and disinfect parts as for a more serious operation. Dilate sphincter muscles well, carry strip of gauze with thread attached above field of operation so as to protect parts from any discharge.

Grasp tumor with artery forceps and pull well down into view. If necessary, apply a second forcep or double tenaculae, then

FIG. I.—1. Beginning of number two plain cat gut suture at upper part of hemorrhoid. 2. Hemorrhoid partly cut off with scissors and surface closed with the over and over stitch following closely as hemorrhoid is removed. 3. Part of hemorrhoid separated. 4. Lower part of hemorrhoid pulled down with forceps to facilitate removal.

commence at the upper end of the pile with a plain No. 2 catgut in a round point curved needle, carry suture around and under the beginning of hemorrhoid, tieing it, taking second hitch with suture to better control hemorrhage before beginning to remove tumor. (See Cut I.) With curved scissors begin cutting at upper part of pile, just below suture, to remove hemorrhoid. Follow up the cutting closely with the suture so that when hemorrhoid is removed the wound is almost completely closed, requiring only two or three stitches with the suture to complete the work, and if properly done there will be practically no loss of blood. (See Fig. II.)

FIG. II. Shows the wound after removal—completely closed with continuous cat gut suture tied at each end. If interrupted suctures are preferred they can be used equally as weli.

Each successive hemorrhoid is removed in the same way, being careful not to remove too much tissue, especially mucous membrane, for fear of some constriction following the operation. It is well sometimes to clip base of tumor on skin surface to prevent removal of too much tissue. If a tag is left at lower end of suture line, it should be clipped off and surface sutured.

It is not necessary to remove all the hemorrhoids deeply, the same result can be secured and save the tissues by carrying the suture deeper in lower part of the tumor—it constricts the blood vessels, lessens blood supply and effectually controls hemorrhoidal condition of rectum.

This is why it is not necessary to remove as much tissue as in other forms of operation, hence less likely to have stricture of rectum.

I have been doing this operation for ten or twelve years, and in that time have had no occasion to use any other method of operating for hemorrhoids. It so far has met all indications. At first, I used a rubber tube wrapped with gauze introduced into rectum after operation, which increased the pain and discomfort to the patient, but by gradually lessening size of dressing I found it was not necessary to use any dressing in rectum, as there was no danger of hemorrhage; patient suffered less and was more comfortable without it.

At completion of operation, the gauze tampon is removed from upper part of rectum and a suppository of Iodoform, Boric Acid and Cocoa Butter introduced and an external dressing applied with firm pressure to part.

Bowels are kept quiet for two or three days, then moved daily by a mild laxative, aided if necessary by sterile oil and boric acid solution thrown into rectum by a soft rubber catheter. After each movement for a week a suppository is introduced to keep parts disinfected.

I formerly used chromacised catgut, but found it was not absorbed as it should be and gave trouble. Number 2 plain catgut lasts a sufficient time, is more pliable and yielding, hence less likely to constrict and cut tissues, No. 1 plain can be used left double.

If a gauze dressing is placed in rectum, the stitches especially chromacised catgut, is likely to hang to the gauze when it is removed and tear the tissues. The advantages of the operation are: ease of execution with few instruments, no loss of blood, no raw surfaces or ulcers left behind, a minimum amount of traumatism, a clean surface and no crushed, charred or strangulated tissue left to slough off, less danger of infection, less pain and a shorter convalescence.

In the last few years, other surgeons have been doing practically the same operation, only modified in a few minor details.

Its advantages certainly justify its use and its results are certainly more satisfactory than the old methods of operating in these cases.

The operation can be done very well under scopolamine, and morphine, with local anasthesia but the divulsion of the sphincter muscle is not as complete and the work can not be completed in detail so well.

The anaesthesia will depend upon the dexterity of the operator, his individual preference, and the condition of the case to be operated upon.

## Figures and Tables

**FIG. I. f1:**
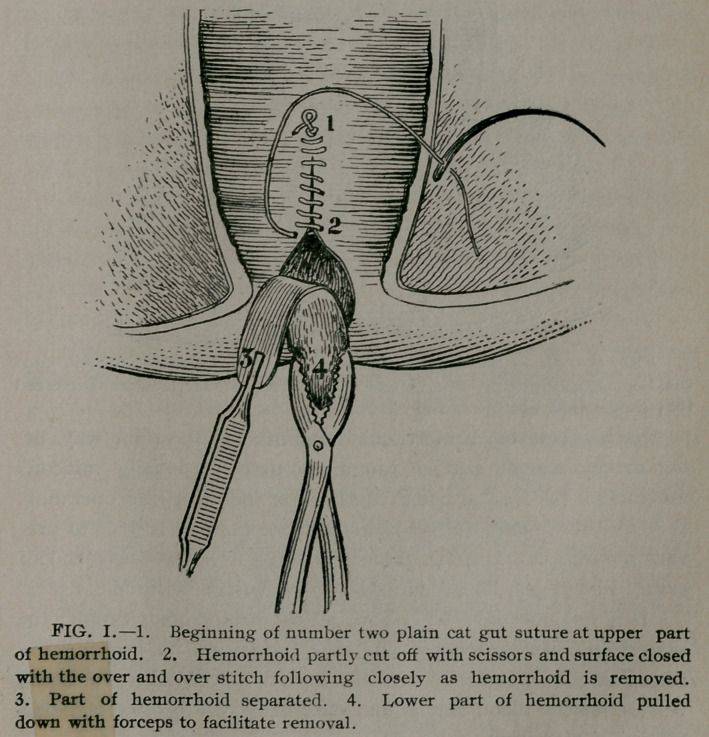


**FIG. II. f2:**